# Notes on the genus *Trigonotoma* from China, with descriptions of two new species (Carabidae, Pterostichinae)

**DOI:** 10.3897/zookeys.921.47258

**Published:** 2020-03-24

**Authors:** Pingzhou Zhu, Hongliang Shi, Hongbin Liang

**Affiliations:** 1 Key Laboratory of Zoological Systematics and Evolution, Institute of Zoology, Chinese Academy of Sciences, Beijing 100101, China Institute of Zoology, Chinese Academy of Sciences Beijing China; 2 College of Life Science, University of Chinese Academy of Sciences, Beijing 100049, China University of Chinese Academy of Sciences Beijing China; 3 College of Forestry, Beijing Forestry University, Beijing 100083, China Beijing Forestry University Beijing China

**Keywords:** character evolution, endophallus, key, Trigonotomina

## Abstract

The genus *Trigonotoma* in China is studied, with descriptions of two new species, *T. digitata***sp. nov.** and *T. constricta***sp. nov.** One species is reported as new to China, *Trigonotoma indica* Brullé, 1834. Species relationships within Chinese *Trigonotoma* are briefly discussed mainly based on the endophallic characters.

## Introduction

*Trigonotoma* is a genus under the subtribe Trigonotomina (Carabidae: Pterostichini) which can be easily recognized by the very short and wide mentum tooth. A total of 51 *Trigonotoma* species has been recorded mainly from Oriental Region (Roux et al. 2016). However, only three species were distributed in China: *T. lewisii* Bates, 1873 widely distributed in east Asia and abundant, *T. dohrni* Chaudoir, 1852 widely distributed in south China but relatively rare, and *T. sinica* Dubault, Lassalle & Roux, 2011 only recorded in Yunnan Province and very rare (Bates 1873, Dubault et al. 2010, Chaudoir 1852, Dubault et al. 2011). Herein, two new species and a new record are proposed.

The taxonomic value of the everted endophallus of Carabidae has been recognized in recent decades, both for systematics and species identification (Shi and Liang 2015, Zhu et al. 2018). Thus, we studied the male endophallus of all available Chinese species (five of six known species, except *Trigonotoma sinica*) and briefly discuss possible relationships of some of the species.

The primary purposes of this paper are to describe two new species of *Trigonotoma*, provide a key for Chinese *Trigonotoma* species determinations, and describe and illustrate the endophallus of five Chinese *Trigonotoma* species (except for *T. sinica*) and discuss their relationships.

## Materials and methods

This paper is based primarily on examination of specimens from China. The majority of specimens examined, including all types of new species, are deposited in the collection of the Institute of Zoology, Chinese Academy of Sciences, Beijing, China (**IZAS**). The specimens examined or cited from other collections are indicated with abbreviations.

**CCCC** Collection of Changchin Chen, Tianjin, China

**MNHN** Muséum National d’Histoire Naturelle, Paris, France

**SNU** Shanghai Normal University, Shanghai, China

**ZSM** Zoologische Staatssammlungen, München, Germany

The body length (**BL**) was measured from apical margin of labrum to elytral apex; the body width (**BW**) was measured along elytral greatest width. The metepisternum length (**ML**) was measured along its outer margin; the basal width (**MW**) was measured along its oblique basal margin (Fig. 13). The pronotum basal width (**PBW**) was measured along its basal margin. For description of the endophallus, all lobes were named based on their homology inferences but not actual locations. The abbreviations used in endophallus are as follows: gonopore (**gp**), gonopore lobe (**gpl**), V-shaped setose area (**sa**), basal band (**bb**), chitinized piece (**cp**), basal lobe (**bl**), apical lobe (**al**), apical lobe-1 (**al-1**), apical lobe-2 (**al-2**), apical lobe-3 (**al-3**), left lobe (**ll**), left basal lobe (**lb**), left basal lobe-1 (**lb-1**), left basal lobe-2 (**lb-2**), left apical lobe (**la**), right lobe (**rl**). Other terms used and methods of measurement, preparation of figures, dissection, and endophallus everting procedures are mainly consistent with what we adopted in our previous work (Shi et al., 2013; Shi & Liang, 2015).

## Taxonomy

### Genus Trigonotoma

Taxon classificationAnimaliaColeopteraCarabidae

Dejean, 1828

FF1609DF-53C3-530B-811E-DEF82B921076

#### Type species.

*Trigonotoma viridicollis* Dejean, 1828 [=*Trigonotoma indica*Brullé 1834]

#### Diagnosis.

Among the six genera (*Trigonotoma* Dejean, 1828, *Lesticus* Dejean, 1828, *Euryaptus* Bates, 1892, *Nesites* Andrews, 1931, *Pareuryaptus* Dubault, Lassalle & Roux, 2008, and *Leiolesticus* Roux, Lassalle & Dubault, 2016) of Trigonotomina, *Trigonotoma* can be distinguished from others in the subtribe by the following character combinations: first antennomere (scape) longer than the lengths of the 2^nd^, 3^rd^, and 4^th^ antennomeres combined; apex of labrum emarginate, with six setae equidistantly placed; mentum notably shortened; parascutellar striae present; third elytral interval without setigerous pore; posterior margin of sternite VII with four setae in females. Detailed descriptions and distributions have recently been provided (Roux et al. 2016).

### Key to Chinese species of *Trigonotoma*

**Table:** 

1	Metepisternum short and wide, length less than or subequal to its basal width (ML/MW<1) (Fig. 13B)	**2**
–	Metepisternum long and narrow, length much greater than its basal width (ML/MW > 1.3) (Fig. 13A)	**3**
2	Pronotum slightly narrowed to the base, very similar to that of *T. lewisii* (PW/PL = 1.27, PW/PBW = 1.55); pronotal basal foveal grooves well defined and separated (Figs 15, 17); male genitalia with the left margin of apical orifice strongly prominent and then deeply notched (Fig. 3)	***T. digitata* sp. nov.**
–	Pronotum strongly widened near middle and constricted to the base (PW/PL = 1.36, PW/PBW = 1.81); pronotal basal fovea with inner and outer grooves vaguely defined, partly fused (Figs 14, 16); male genitalia with the left margin of apical orifice gently sinuate near middle (Fig. 7)	***T. constricta* sp. nov.**
3	Pronotum lateral margins strongly sinuate before posterior angles, posterior angles pointed (Fig. 18)	***T. indica* Brullé**
–	Pronotum lateral margins not or only weakly sinuate before posterior angles, posterior angles rounded (Figs 19–21)	**4**
4	Pronotum completely black, not metallic	***T. sinica* Dubault, Lassalle & Roux, 2010**
–	Pronotum greenish to cupreous metallic	**5**
5	Pronotum with dense and coarse punctures in the middle-basal area between basal fovea (Fig. 21); pronotum weakly narrowed to the base	***T. dohrni* Chaudoir, 1852**
–	Pronotal base completely glabrous (Fig. 20), or with a few punctures restricted in the basal fovea area, the middle region between basal fovea completely glabrous (Fig. 19); pronotum distinctly narrowed to the base	***T. lewisii* Bates, 1873**

### Trigonotoma digitata sp. nov.

Taxon classificationAnimaliaColeopteraCarabidae

867A11B5-969D-5B63-8A8F-F593C30E2478

http://zoobank.org/8B91DE0C-06A8-41CD-80DB-578B369A1E7B

Figures 1–4

#### Type locality.

Guangdong: Xinfeng, Yunji Mountain (24.12N, 114.16E), altitude 1318 m.

#### Type material.

***Holotype***: Male (IZAS), BL = 17.2 mm, board mounted, genitalia preserved in 100% ethanol in a microvial pinned under specimen, “China, Guangdong, Xinfeng, Yunji Mountain, pitfall trap, 24.115841N, 114.163535E”; “1318 m, 2017.V.20–25, Liu Y. Z. & Yu S. P. lgt., Institute of Zoology, CAS, Yunji Mountain, Xinfeng”; “HOLOTYPE ♂ *Trigonotoma digitata* sp. nov., des. ZHU & SHI 2019” [red label].

#### Diagnosis.

Dorsal side bicolored, with strong metallic luster, pronotum cupreous green, elytra dark purple; pronotum slightly narrowed to the base; posterior angles completely rounded; pronotal base including the basal fovea completely glabrous; basal fovea with inner and outer grooves well defined; metepisternum short and wide, length subequal to its basal width; median lobe of aedeagus strongly lobed and notched on the left margin.

#### Comparison.

The new species is different from all other known species of *Trigonotoma* by its distinct male genitalia (Fig. 3). At first glance, the new species is very similar to *T. lewisii* in external appearance, but these two species can be readily distinguished by the differences of metepisternum and male genitalia.

**Figures 1–4.**
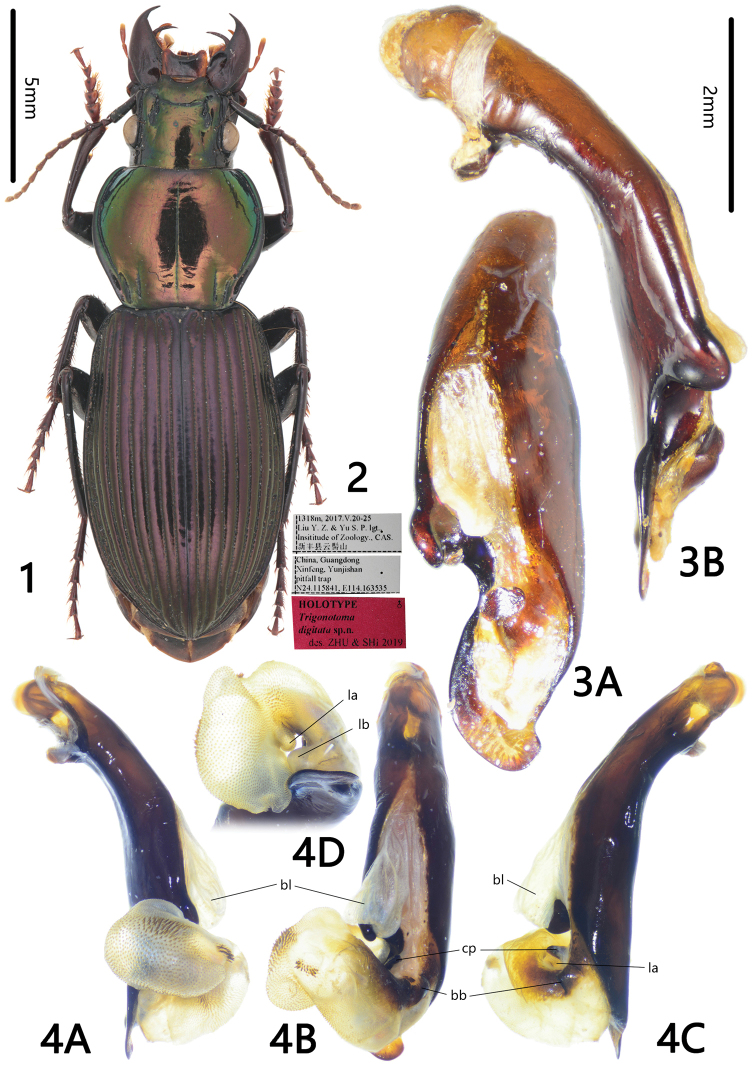
*Trigonotoma digitata* sp. nov. **1** Habitus of holotype (male, Guangdong, IZAS) **2** labels of holotype **3** median lobe of aedeagus (holotype) **A** dorsal view **B** left lateral view **4** endophallus (holotype) **A** left lateral view **B** dorsal view **C** right lateral view **D** apical view.

#### Description.

BL = 17.2 mm, BW = 6.2 mm. Dorsal side bicolored with strong metallic luster: head and pronotum cupreous green, elytra purple; appendages dark, antennomeres 2–11, labial and maxillary palpi, apex of mouthparts and tarsomeres dark brown; ventral side black, without metallic luster. Head and pronotum with isodiametric microsculpture and minute punctures; elytra with transversal microsculpture.

***Head*** with vertex smooth; frontal impressions deep and straight, longitudinally extending to the level of midpoint of eyes; labrum and clypeus both with anterior margins deeply emarginate; temporae straight, not swollen behind eyes; antennae reaching pronotum basal quarter.

***Pronotum*** slightly transverse, PW/PL = 1.24, widest near anterior third; lateral margins curved in middle, and then gently narrowed to base, PW/PBW = 1.55; lateral margins straight in front of posterior angles, posterior angles rounded, not forming distinct angle; anterior margin straight, anterior angles widely rounded; posterior margin of nearly same width as anterior margin, gradually extended backward at lateral sides; disc completely glabrous, gently convex; median line fine but clearly defined, almost reaching posterior margin. Basal fovea deep and glabrous, without puncture or wrinkle; inner and outer grooves well defined, region between them deeply depressed, inner groove straight, slightly longer than curved outer groove.

***Elytra*** oviform, EL/EW = 1.63, widest near posterior third; basal ridge complete, curved at fourth interval; humeral angles rounded, without teeth; intervals fairly convex, striae deeply incised, with coarse punctures; parascutellar striae short, apex almost conjunct to first stria; parascutellar pore present; third interval without setigerous pore; umbilicular series on ninth interval composed of approximately 25 pores, sparse in middle.

***Ventral side***: Propleuron and mesoepisternum with sparse and coarse punctures; metepisternum short and wide, ML/MW = 1.02, with sparse and coarse punctures; abdominal sternites glabrous on middle, with a few coarse punctures on lateral sides of sternites II and III, and shallow wrinkles on lateral sides of all sternites.

***Legs***: Metatarsomeres I and II strongly carinate on basal 3/4 of outer surface, distinctly carinate on basal half of mesotarsomere I and metatarsomere III; fifth tarsomeres of all legs with three or four pairs of spines ventrally.

***Median lobe of male genitalia*** bent approx. 45° (the included angle between apical lamella and axes of basal portion of aedeagus). Apical orifice long and sinuate, constricted in middle, opened dorsally, and slightly turned to left. In dorsal view, right margin of aedeagus straight, and then sinuate before apical lamella; left margin with a digitiform lobe near midpoint of apical orifice, deeply notched anterior to lobe, and then widely arched reaching apex of apical lamella; apex of digitiform lobe rounded and bent to dorsal side; apical lamella short and wide, length approx. half its basal width; strongly bent to right, apex truncated, without tooth; dorsal surface without ridge.

***Endophallus*** (Fig. 4) rotated to dorsal-left direction of aedeagus, major portion of endophallus on dorsal side of aedeagus; gpl folded so, invisible in Fig. 4; bb elongated, extended from apical orifice to middle part of endophallus; cp at left margin of apical orifice. Three distinct lobes recognized: bl moderately large, slightly prolonged, located at base of apical orifice, pointing to apical direction of aedeagus, membranous, without scales; lb small, rounded, located at base of endophallus and left side of apical orifice, pointing to left basal direction of aedeagus; la smaller than lb, rounded, located at left side of endophallus, with fine scales. Apex of endophallus large, elongate, with heavy spines on central and basal surfaces, and fine scales on other areas.

#### Distribution.

Yunji Mountain, Xinfeng, Guangdong. Only known from the holotype.

#### Etymology.

The specific epithet *digitata* is based on the Latin for finger and indicates the finger-shaped lobe on the aedeagus of the males. It is treated as an adjective in the nominative singular.

### Trigonotoma constricta sp. nov.

Taxon classificationAnimaliaColeopteraCarabidae

C19CE897-B484-592B-862A-0CCDA26AF888

http://zoobank.org/25E4489B-A3BF-4FCD-8F2F-C0E8A954F79C

Figures 5–8

#### Type locality.

Hunan: Guidong, Bamian Shan Mt. (25.99N, 113.71E), altitude 1510 m.

#### Type material.

***Holotype***: Male (IZAS), BL = 15.8 mm, board mounted, genitalia preserved in glycerin in a microvial pinned under specimen, “China, Hunan Guidong Co. Bamian Shan Mt., 25°59'33"N, 113°42'25"E, mixed forest, shrub, flower sifted & beating, ca. 1510m, 01.VI.2014, Peng, Shen, Yu & Yan”; “LX-5-1-1-8466”; “HOLOTYPE ♂ *Trigonotoma constricta* sp. nov., des. ZHU & SHI 2019” [red label].

#### Diagnosis.

Dorsal side bicolored, pronotum metallic dark green, elytra dark purple; pronotum strongly narrowed to the base; posterior angles obtuse-rounded; pronotal base including the basal fovea completely glabrous; basal fovea with inner and outer grooves vaguely defined, anterior half separated from each one, posterior half fused together; metepisternum short and wide, length subequal to its basal width.

#### Comparisons.

This new species can be readily distinguished from all other *Trigonotoma* from China by the narrowly constricted pronotum base. *Trigonotoma concinna* from Java has the pronotum shape and basal fovea very similar to *T. constricta*, but differs by its larger size (19–21 mm), longer metepisternum, and longer apical lamella of the aedeagus.

**Figures 5–8.**
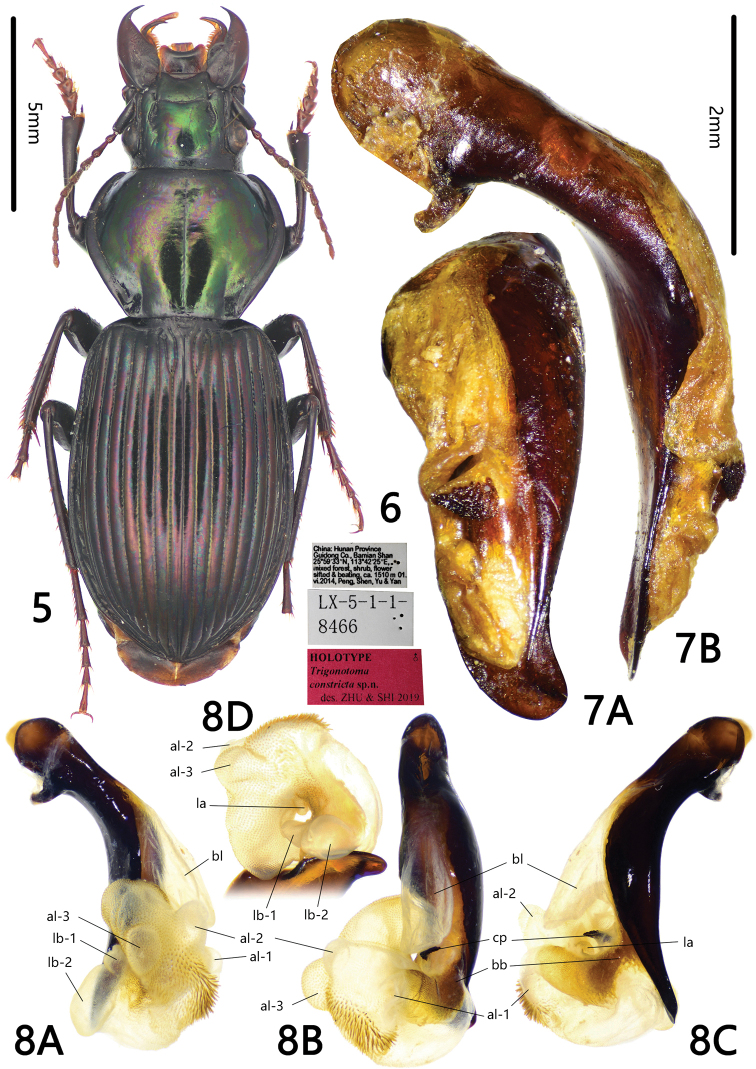
*Trigonotoma constricta* sp. nov. **5** Habitus of holotype (male, Hunan, IZAS) **6** labels of holotype **7** median lobe of aedeagus (holotype) **A** dorsal view **B** left lateral view **8** endophallus (holotype) **A** left lateral view **B** dorsal view **C** right lateral view **D** apical view.

#### Description.

BL = 15.8 mm, BW = 5.9 mm. Dorsal side bicolored with strong metallic luster: head and pronotum dark green, elytra purple; appendages dark, antennomeres 2–11, labial and maxillary palpi, apex of mouthparts and tarsomeres dark brown; ventral side black, without metallic luster. Head and pronotum with isodiametric microsculpture and minute punctures; elytra with transversal microsculpture.

***Head*** with vertex smooth; frontal impressions deep and straight, longitudinally extending to the level of midpoint of eyes; labrum and clypeus both with anterior margins deeply emarginate. Temporae straight, not swollen behind eyes; antennae reaching pronotum basal quarter.

***Pronotum*** wide and round, PW/PL = 1.39, widest slightly before middle; lateral margins strongly widened and curved near middle, and then strongly constricted to base, PW/PBW = 1.81; lateral margins straight before posterior angles, posterior angles obtuse, forming indistinct angles; anterior margin straight, anterior angles widely rounded; posterior margin with width approximately equal to that of anterior margin, very slightly extended backward at lateral sides. Disc completely glabrous, gently convex; median line fine but clearly defined in middle, gradually shallowed, reaching neither posterior nor anterior margin; basal fovea deep and glabrous, without puncture or wrinkle; inner and outer grooves vaguely defined, partly fused together, region between them deeply depressed, so that basal fovea forms simple depressions.

***Elytra*** oviform, EL/EW = 1.54, widest near posterior third; basal ridge complete, sinuate at inner half; humeral angles rounded, without tooth, intervals fairly convex; striae deeply incised, with fine punctures; parascutellar striae short, apex conjunct to first stria; parascutellar pore present; third interval without setigerous pore; umbilicate series on ninth interval composed of approximately 25 pores, sparse in middle.

***Ventral side***: Propleuron glabrous, mesoepisternum with dense and coarse punctures; metepisternum short and wide, ML/MW = 0.99, with dense and coarse punctures; abdominal sternites glabrous on middle, with a few coarse punctures on lateral sides of sternites II and III, and shallow wrinkles on lateral sides of all sternites.

***Legs***: Metatarsomeres I and II strongly carinate almost along their full length of outer surface, very shallowly carinate on basal half of mesotarsomere I and metatarsomere III; fifth tarsomeres of all legs with three or four pairs of spines ventrally.

***Median lobe of male genitalia*** bent approximately 60° (the included angle between apical lamella and axes of basal portion of aedeagus). Apical orifice long and wide, reaching basal fourth of aedeagus, opened dorsally, slightly turned to left; right margin of apical orifice straight, left margin gently sinuate and notched near middle. In lateral view, aedeagus apex slightly bent downwards; ventral margin almost straight; apical lamella slightly thickened near base. In dorsal view, aedeagus apex broadly bent to right side; apical lamella length subequal to its basal width, with an indistinct oblique ridge, apex rounded-truncate, without tooth.

***Endophallus*** (Fig. 8) rotated to dorsal-left direction of aedeagus, major portion of endophallus on dorsal side of aedeagus; gpl folded so, invisible in Fig. 8, bb short, not reaching middle part of endophallus; cp at left margin of apical orifice. Seven distinct lobes recognized: bl moderately large, slightly prolonged, located at base of apical orifice, pointing to apical direction of aedeagus, membranous, without scales; lb-1 small, rounded, located at base of endophallus and left side of apical orifice, pointing to left basal direction of aedeagus, without decorations; lb-2 larger than lb-1, elongate, located at right side of lb-1, pointing to left apical direction of aedeagus, without decoration; la smaller than lb-1, rounded, located at left side of endophallus, with fine scales; al-1 small, rounded, located at right basal side of endophallus, without decoration; al-2 slightly larger than al-1, rounded, located at right apical side of endophallus, decorated with very fine scales; al-3 with same size as al-2, rounded, located at left apical side of endophallus, decorated with fine scales. Apex of endophallus large, elongate, with a list of heavy spines on central surface, and fine scales on other area.

#### Distribution.

Bamian Shan Mt., Guidong, Hunan. Only known from the holotype.

#### Etymology.

The specific epithet *constricta* refers to the narrowly constricted base of the pronotum. It is treated as an adjective in the nominative singular.

### Trigonotoma indica

Taxon classificationAnimaliaColeopteraCarabidae

Brullé, 1834 new record

2CA500C9-F07E-5547-B181-5A37B23AED5E

Figures 9–12

Brullé, 1834: 333 (Original: Trigonotoma, type in MNHN; type locality: Bengale); Chaudoir, 1868: 158; Bates, 1886: 145; Csiki, 1929: 517; Andrewes, 1930: 354; Andrewes, 1938: 138; Morvan, 1994: 328; Lorenz, 2005: 895; Dubault et al., 2007: 210; Kirschenhofer, 2007: 8; Dubault et al., 2008: 179; Roux et al., 2016: 122; Löbl I & Löbl D, 2017: 755.
viridicollis Dejean, 1828: 183, (Original: Trigonotoma; type in MNHN; type locality: India); Guérin-Méneville, 1829:44; Andrews, 1919: 148. Unavailable name, misidentification of Omaseus viridicollis Macleay, 1825. (Synonym)
baehri Kirschenhofer, 1997: 700, (Original: Trigonotoma; type in ZSM; type locality: C-Indien, MPR. Panna, Nat. Park). (Synonym)

#### Type locality.

Bangladesh.

#### Material examined.

1 male (IZAS), “China, Tibet, Mêdog, Baibung Township, 780 m, 2011.VIII.10–13, Bi W. X.”; 1 female (CCCC), “China, Tibet, Mêdog, Baibung Township, 700 m, 2011.VIII.09, YANG X. D. Leg. B11y2633, CCCC”; 1 female (IZAS), “Mêdog, light trap, 2016.VIII.5, Qiu T. F.”; 18 males and 30 females (CCCC), “India, Andhra Pradesh Nellore District, Naidupet Mandal, Dwarakauram vili., 2010.IX.11–X.3, Chen C. C. Leg.”.

#### Diagnosis.

BL = 20mm. Dorsal side bicolored, pronotum with metallic luster, purple, green, blue or nearly black, elytra black with faint metallic reflections; pronotum lateral margins strongly sinuate in front of posterior angles; posterior angles sharp and base rectangular; basal fovea more or less punctate and rugose; metepisternum long and narrow; apical lamella of aedeagus with rounded apex, shallowly notched or not. *Trigonotoma indica* can be readily distinguished from all other Chinese species by the pronotum lateral margins that are strongly sinuate near base.

#### Supplementary descriptions on endophallus.

Endophallus (Fig. 12) bent to dorsal direction of aedeagus, major portion of endophallus on dorsal side of aedeagus; gp located at approx middle of aedeagus, oriented to aedeagal base; gpl large, rounded, membranous, bb absent; cp absent. Four distinct lobes recognized: bl moderately large, slightly prolonged, located at base of apical orifice, pointing to apical direction of aedeagus, with a few scales on left side; ll moderately large, divided into several sub-lobes, located at left side of endophallus, with fine scales; rl smaller than ll, rounded, located at right side of endophallus, with fine scales; al large, divided into several sub-lobes, located at apex of endophallus, connected with base of endophallus through a narrow area, forming a dumbbell-shape, with fine scales. Middle of endophallus large, rounded, with a V-shaped sa on left middle and fine scales on apex.

**Figures 9–12.**
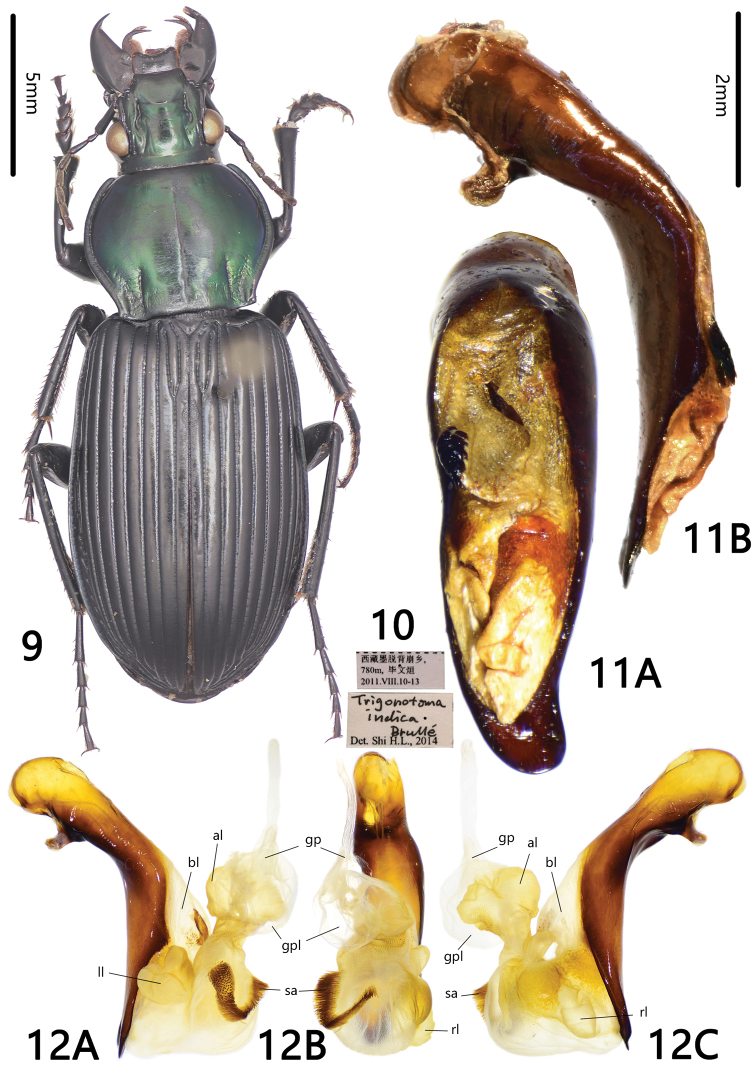
*Trigonotoma indica* Brullé, 1834. **9** Habitus (male, Xizang, IZAS) **10** labels **11** median lobe of aedeagus **A** dorsal view **B** left lateral view **12** endophallus **A** left lateral view **B** dorsal view **C** right lateral view.

#### Distribution.

China (Tibet: Mêdog), India, Bangladesh, Sri Lanka, and Pakistan. Another subspecies, *T. indica nepalensis*, is distributed in Nepal.

#### Remarks.

It is expected this species would be found to be widely distributed in and around China. Identification is based on the comparison of the image of holotype (Roux et al., 2016) and specimens from Andhra Prad., India. Compared with the specimens from India, *T. indica* from Mêdog is slightly larger and more vividly green on its pronotum.

## Discussion

Before the present study, three *Trigonotoma* species were recorded from China. Here, we add three more species bringing the total number of Chinese *Trigonotoma* to six. Preliminary conclusions on species relationships within Chinese *Trigonotoma*, mainly based on the endophallic characters, are presented below.

**Figures 13–21.**
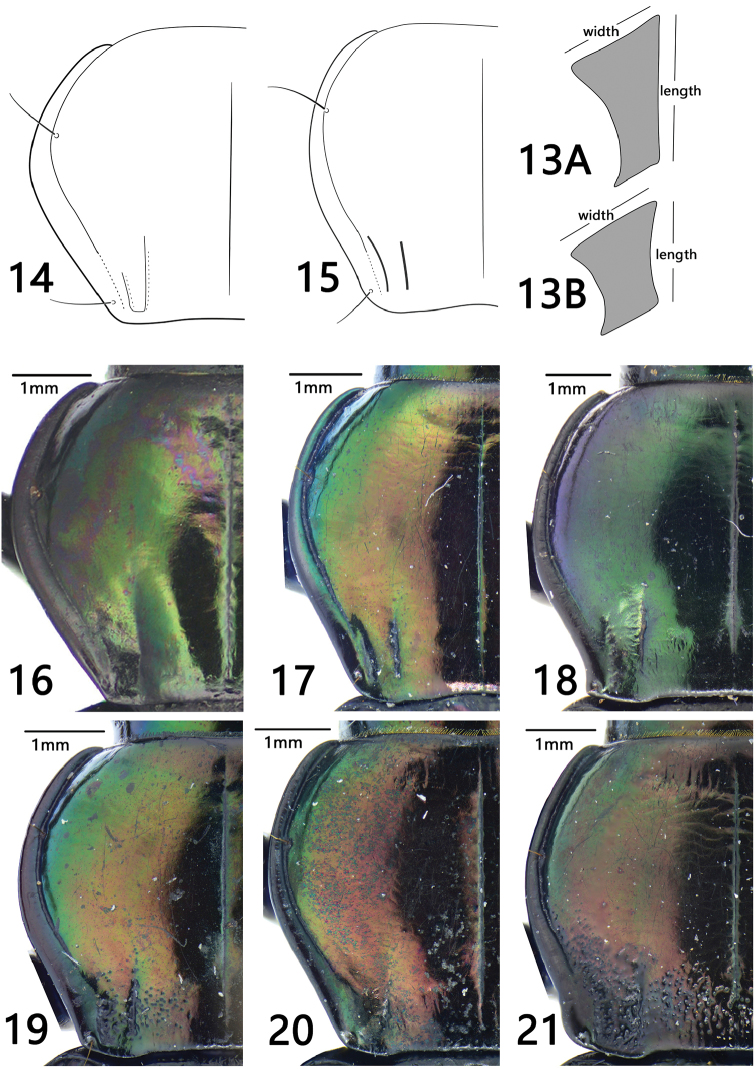
**13** Metaepisternum **A***Trigonotoma lewisii* (long and narrow) **B***T. constricta* (short and wide) **14–21** pronotum **14, 16***T. constricta***15, 17***T. digitata***18***T. indica***19, 20***T. lewisii***21***T. dohrni*.

The endophallus characters of *T. constricta* and *T. digitata* are very similar, sharing the same bb, cp, bl, la and dorsal-left rotation. Their main differences are that in *T. digitata*, lb is divided into lb-1 and lb-2 and three apical lobes (al-1, al-2 and al-3) appear on apex of endophallus, which makes it more complex than in *T. constricta*. In addition to characteristics of the endophallus, the short metepisternum is another important shared character state. As we discussed in the previous paper (Zhu et al., 2018), the shape of metepisternum has important taxonomic value in *Lesticus*. The shortened metepisternum is apomorphic in *Trigonotoma*, similar to what is found in *Lesticus* and several groups of Carabidae. The two species described here are the first known with short metepisternum from China. Among all *Trigonotoma*, only five other species are known to have this character: *Trigonotoma morvani* Deuve & Lassalle, *T. himalchuliensis* Lassalle (Nepal), *T. cylindriceps* Straneo (India), *T. igneicollis* Bates (Myanmar), and *T. buehleri* Straneo (Indonesia, Sumba). The two newly described Chinese species are hypothesized to be closely related, possibly sister species, based on the character of metepisternum, the shared dorsal-left curled endophallus, and the adjacent distributions.

**Figures 22, 23.**
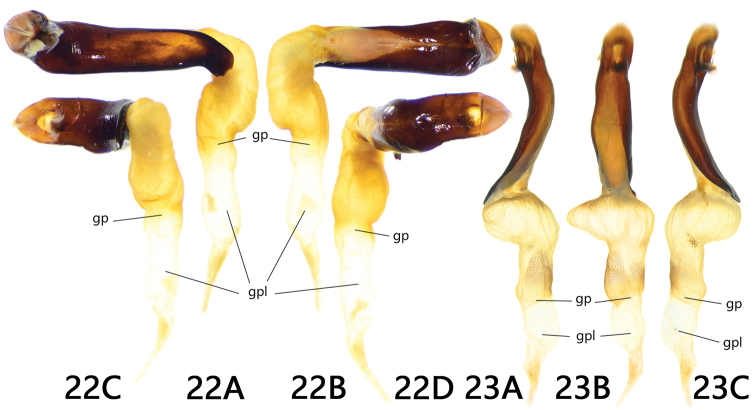
Endophallus**22***Trigonotoma dohrni***A** ventral view **B** dorsal view **C** apical view **D** basal view **23***T. lewisii***A** left lateral view **B** dorsal view **C** right lateral view.

**Figure 24.**
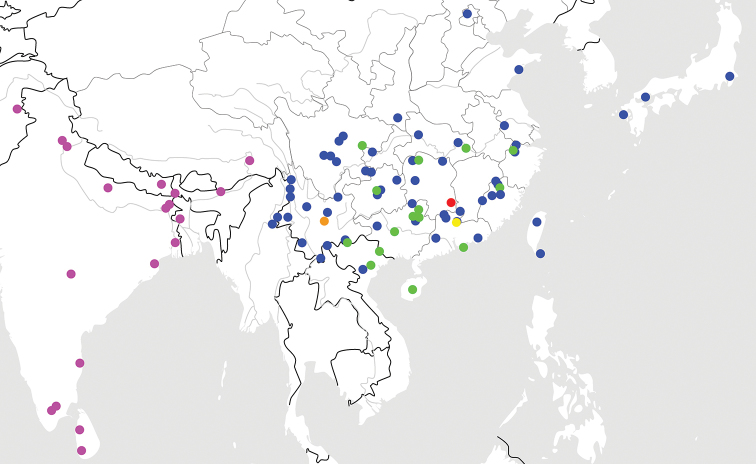
Distribution map for *Trigonotoma* from China: *T. constricta* sp. nov. (red); *T. digitata* sp. nov. (yellow); *T. sinica* Dubault, Lassalle & Roux (orange), the precise locality was not mentioned in the original literature; *T. dohrni* Chaudoir (green); *T. lewisii* Bates (blue); *T. indica* Brullé, 1834 (purple).

Two previously described species were also studied. The endophallus of *T. lewisii* and *T. dohrni* show extensive similarity but are quite different from *T. digitata* and *T. constricta*. They have a shared character of a prolonged, straight, and nearly glabrous endophallus without any lobe, scale, setose, band, or chitinized piece. Moreover, bl, cp, and bb are also absent. These two species are different from each other in the orientation of endophallus: *T. lewisii* extending to genital apex, slightly deflected to dorsum, gonopore oriented to aedeagal apex (Fig. 23), while *T. dohrni* markedly deflexed to the right, forming a right angle with aedeagus, and gonopore oriented to the right side of aedeagus (Fig. 22). As to the external characters, they have completely different pronotal form but similar long metapisternum. Additionally, they are both widely distributed in south China, Myanmar, and Vietnam, while *T. lewisii* is also distributed in north China, Korea, and Japan (Fig. 24). In conclusion, a close relationship of these two species is possible.

The endophallus of *T. sinica* has not been examined.

*Trigonotoma indica* is different from the species discussed above in terms of endophallus characters, external characters, and distribution. The endophallus of *T. indica* is bent in the dorsal direction and the gonopore is oriented toward the aedeagal base. In addition, cp and bb are absent, but a V-shaped sa appears on the left middle of endophallus. And the posterior angle is pointed and forms a right angle in *T. indica*. In all other Chinese *Trigonotoma*, however their pronotum lateral margin differs, it is not pointed. Outside of the Chinese fauna, there are five other species with pointed posterior angles: *T. oberthuri* Tschitscherine, *T. tenebrosa* Dubault et al., *T. cylindriceps* Straneo, *T. morvani* Deuve & Lassalle, and *T. himalchuliensis* Lassalle, all from Indian fauna. Additionally, the endophallus of the first two are bent in a dorsal direction and the gonopore is oriented towards the aedeagal base (Roux et al., 2016), the same as *T. indica*. Finally, considering the different distribution patterns and morphological characters, *T. indica* could be related to the above Indian species and distant from other four Chinese species.

## Supplementary Material

XML Treatment for Trigonotoma

XML Treatment for Trigonotoma digitata

XML Treatment for Trigonotoma constricta

XML Treatment for Trigonotoma indica

## References

[B1] Andrewes HE (1919) On the types of Oriental Carabidae in the British Museum, and in the Hope Department of the Oxford University Museum. The Transactions of the Entomological Society of London 1919: 119–217. 10.1111/j.1365-2311.1919.tb00006.x

[B2] Andrewes HE (1930) Catalogue of Indian Insects (Part 18: Carabidae). Calcutta: Government of India Central Publication Branch.

[B3] Andrewes HE (1931) Papers on Oriental Carabidae – XXV. The Annals and Magazine of Natural History (10) 7: 513–528. 10.1080/00222933108673342

[B4] Andrewes HE (1938) Papers on Oriental Carabidae – XXXV. On the types of some Indian Genera. The Annals and Magazine of Natural History (11)3: 128–139. 10.1080/03745481.1939.9723582

[B5] Bates HW (1873) On the Geodephagous Coleoptera of Japan. The Transactions of the Entomological Society of London 1873: 219–322. 10.1111/j.1365-2311.1873.tb00643.x

[B6] Bates HW (1886) On the Geodephagous Coleoptera collected by Mr. George Lewis in Ceylon. The Annals and Magazine of Natural History (5)17: 68–212, 214–221. 10.1080/00222938609460134

[B7] Bates HW (1892) Viaggio di Leonardo Fea in Birmania e regioni vicine. XLIV. List of the Carabidae. Annali del Museo Civico di Storia Naturale di Genova 32: 267–428.

[B8] Brullé A (1834) Pp. 1–240. In: Audouin JV, Brullé GA (Eds) Histoire naturelle des insectes, traitant de leur organisation et de leurs mœurs en général, et comprenant leur classification et la description des espèces. Tome IV. Coléoptères. I. F.D. Pillot, Paris, 8 + 479 pp. [1834–1835].

[B9] Chaudoir M (1852) Mémoire sur la famille des carabiques. 3e partie. Bulletin de la Société Impériale des Naturalistes de Moscou 25(1): 3–104.

[B10] Chaudoir M (1868) Révision des Trigonotomides. Annales de la Société Entomologique de Belgique 11[1867–68]: 151–165.

[B11] Csiki E (1929) Carabidae: Harpalinae III (Pars 104). In: Junk W, Schenkling S (Eds) Coleopterorum catalogus. Volumen II. Carabidae II. W. Junk, Berlin, 1022 pp.

[B12] Dejean PFMA (1828) Species général des coléoptères, de la collection de M. le Comte Dejean. Tome troisième. Méquignon-Marvis, Paris, vii + 556 pp.

[B13] Dubault G Lassalle B Roux P (2007) Types of ‘Trigonotomi’ of the Collections of the Museum National D’histoire Naturelle, in Paris (Coleoptera, Pterostichidae). Bulletin de la Societe Entomologique de France 112: 209–222.

[B14] Dubault G Lassalle B Roux P (2008) Les genres des “Trigonotomi”: *Pareuryaptus* n. gen. et révision des *Euryaptus* Bates, 1892 (Coleoptera, Pterostichidae). Bulletin de la Société Entomologique de France 113: 239–248.

[B15] Dubault G Lassalle B Roux P (2010) A propos de *Trigonotoma lewisi* Bates, 1873 (Coleoptera, Pterostichidae, Trigonotomi). Le Coléoptériste 13(2): 115–128.

[B16] Dubault G Lassalle B Roux P (2011) Nouvelles espèces de *Trigonotoma* (Coleoptera, Pterostichidae, Trigonotomi). Le Coléoptériste 13: 187–199.

[B17] Guérin-Méneville FÉ (1829) Iconographie du règne animal de G. Cuvier, ou représentation d’après nature de l’une des espèces les plus remarquables, et souvent non encore figurées, de chaque genre d’animaux. Avec un texte descriptif mis au courant de la science. Ouvrage pouvant servir d’atlas à tous les traités de zoologie. Paris: J.B. Baillière [1828–1837], 450 pls. 10.5962/bhl.title.6255

[B18] Kirschenhofer E (1997) Neue Arten der Gattungen *Pterostichus* Bonelli, 1810, *Synuchus* Gyllenhal, 1810, *Lesticus* Dejean, 1828 und *Trigonotoma* Dejean, 1828 aus Ost- und Südostasien (Coleoptera, Carabidae: Pterostichinae). Linzer Biologische Beiträge 29: 689–714.

[B19] Kirschenhofer E (2007) Taxonomische Bemerkungen zu den Gattungen *Lesticus* Dejean, 1828 und *Trigonotoma* Dejean, 1828 mit Beschreibung sieben neuer Taxa (Coleoptera: Carabidae). Koleopterologische Rundschau 77: 1–16.

[B20] Löbl I Löbl D (2017) Catalogue of Palaearctic Coleoptera, Volume 1: Archostemata – Myxophaga – Adephaga. E J Brill, Denmark, 1477 pp 10.1163/9789004330290_003

[B21] Lorenz W (2005) Systematic List of Extant Ground Beetles of the World (InsectaColeopteraGeadephaga: Trachypachidae and Carabidae incl. Paussinae, Cicindelinae, Rhysodinae), Second Edition. Wolfgang Lorenz, Tutzing, Germany, 530 pp.

[B22] Macleay WS (1825) Number 1. of Annulosa Javanica, or an Attempt to Illustrate the Natural Affinities and Analogies of the Insects Collected in Java by Thomas Horsfield, M.D. F.L. & G.S. and Deposited by Him in the Museum of the Honorable East-India Company. Kingsbury, Parbury & Allen, London, xii + 50 pp. [1 pl.] 10.5962/bhl.title.65151

[B23] Morvan P (1994) Carabidae nouveaux du Népal et de Malaisie (Coleoptera, Carabidae). Bulletin de la Société Entomologique de France 99: 323–334.

[B24] Roux P Lassalle B Dubault G (2016) Les Trigonotomi. Révision. B. Lassalle et P. Roux, France, 569 pp.

[B25] Shi HL Liang HB (2015) The Genus *Pterostichus* in China II: The Subgenus Circinatus Sciaky, a Species Revision and Phylogeny (Carabidae, Pterostichini). ZooKeys 536: 1–92. 10.3897/zookeys.536.5982 PMC4714036 26798233

[B26] Shi HL Sciaky R Liang HB Zhou HZ (2013) A new subgenus Wraseiellus of the genus *Pterostichus* Bonelli (Coleoptera, Carabidae, Pterostichini) and new species descriptions. Zootaxa 3664(2): 101–135. 10.11646/zootaxa.3664.2.1 26266293

[B27] Zhu PZ Shi HL Liang HB (2018) Four New Species of *Lesticus* (Carabidae, Pterostichinae) from China and Supplementary Comments on the Genus. ZooKeys 782: 129–162. 10.3897/zookeys.782.27187 PMC6160756 30275722

